# Post-dispensing stability surveillance: Stavudine

**DOI:** 10.4102/phcfm.v1i1.14

**Published:** 2009-05-27

**Authors:** Kamsaladevi K. Naidoo, Thavendran Govender, Rellissa A. Deonunan, Terusha Govender, Calveni Naidoo, Ramona Moodley, Melissa Govender, Khahliso P. Miya

**Affiliations:** 1 School of Pharmacy and Pharmacology, University of KwaZulu-Natal, South Africa

**Keywords:** drug dispensing, pharmacy, stavudine, drug storage, HIV/AIDS

## Abstract

**Background:**

Stavudine, a thymidine nucleoside, is a reverse transcriptase inhibitor, which is extensively used in the treatment of HIV infected patients. According to the World Health Organization (2006), stavudine must be stored in well closed containers and be protected from light. In addition, the manufacturer recommends that stavudine be stored below 25°C in a tightly closed container. However, because of the stigma associated with the disease condition, patients may attempt to hide their medication by storing the drug in more anonymous packaging, which may not comply with these storage requirements. Furthermore, the high temperature and humidity conditions found in sub-tropical areas such as Durban, KwaZulu-Natal, place additional environmental stress on the drug. Research has shown that stavudine can degrade to thymine under hydrolytic, oxidative and photolytic conditions. Therefore, this study investigated the stability of stavudine in packaging other than that used by the manufacturer and under temperature and humidity conditions that were higher than those recommended by the manufacturer.

**Method:**

Stavudine capsules were placed in different types of packaging and then subjected to different temperature and humidity conditions. At two week intervals the capsules were analysed using HPLC (high pressure liquid chromatography).

**Results:**

Stavudine capsules stored in packaging other than that used by the manufacturer and under temperature and humidity conditions that are higher than those recommended by the manufacturer showed significant degradation.

**Conclusion:**

Patients taking stavudine stored under sub-optimal conditions may ingest less than the required dose of stavudine. This can lead to drug resistance and treatment failure.

## INTRODUCTION

HIV and AIDS are two of the main challenges facing South Africa today. It is estimated that of the 39.5 million people living with HIV worldwide in 2006, more than 63% were from sub-Saharan Africa. In 2005 about 5.54 million people were estimated to be living with HIV in South Africa^1^.

The first line regimen for ARV naïve adult patients in South Africa comprises of: stavudine, lamivudine, and efavirenz^2^. Stavudine is chemically a thymine nucleoside with inhibitory activity against reverse transcriptase of the human immunodeficiency virus^3^.

Research has found that stavudine can degrade to thymine under hydrolytic, oxidative and photolytic conditions^4^. According to the World Health Organisation^5^, stavudine must be stored in a well closed container protected from light. The manufacturer also recommends that stavudine be stored in tightly closed containers below 25°C ^6^. However, literature has indicated that due to the stigma associated with the disease, stavudine may not always be stored by the patient under these conditions, which may cause stavudine to be stored under conditions that can result in its hydrolysis, oxidation or photolysis. The degradation of stavudine can be especially problematic in sub-tropical and areas of high humidity, such as Durban, KwaZulu-Natal. Therefore, this study investigated the stability of stavudine in packaging other than that used by the manufacturer and under temperature and humidity conditions that are higher than those recommended by the manufacturer.

Stavudine can be quantitatively determined, analytically, by high pressure liquid chromatography (HPLC) which is a precise, accurate, robust and inexpensive method of analysis. This technique allows for the monitoring of the stability of pure drug substances, drugs in formulations and quantification of degradation products^7^. Therefore, this was the analytical technique of choice for this study.

### Background

**FIGURE 1:**
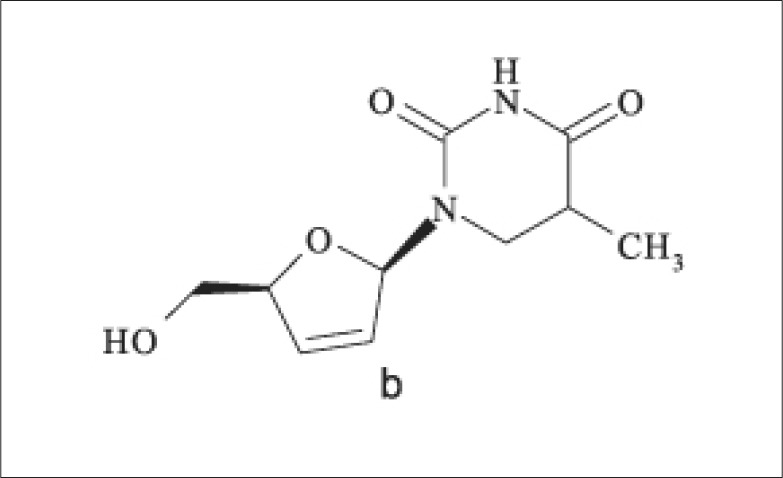
Structure of stavudine^8^

Stavudine is chemically a thymidine nucleoside with inhibitory activity against reverse transcriptase of the human immunodeficiency virus^3^. Its structure is shown in Figure 1.

Stavudine is one of the most widely used substances in the treatment of the acquired immunodeficiency syndrome^8^. The South African National Antiretroviral Treatment Guidelines include stavudine in its first line treatment regimen^2^.

The important structural feature in stavudine that can be considered to be responsible for hydrolytic cleavage of the drug to thymine under acidic, neutral and alkaline conditions is the aminal functionality. The aminal nitrogen can bear a positive charge in acidic conditions as a result of protonation of the thymine moiety, forming an enol, which acts as a better leaving group, thus, assisting in hydrolytic cleavage.

**FIGURE 2:**
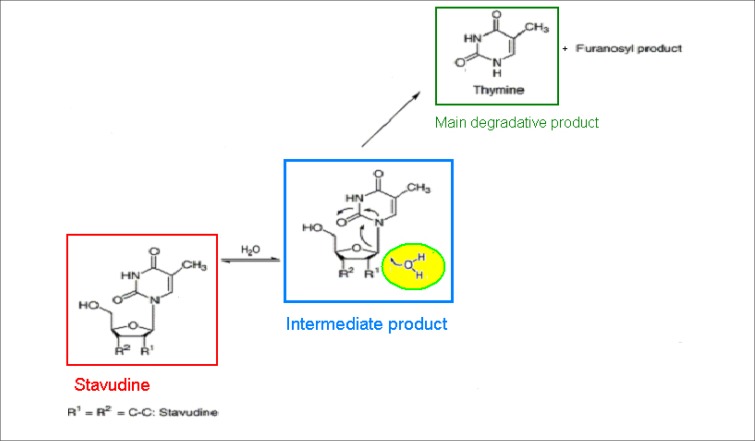
The hydrolytic degradation mechanism of stavudine to its main degradative product, thymine^4^

The mechanism of degradation of stavudine is described in Figure 2.

Stavudine may degrade to thymine under hydrolytic, oxidative and photolytic conditions^4^. Furthermore other research has shown that stavudine capsules stored at 50°C/90% RH, presented more than a ten fold increase in thymine, the degradation product of stavudine during the 90 day study period^8^.

In Durban, South Africa, the highest recorded temperature for 2006 was 42°C and the highest relative humidity experienced was 80%, while the mean recorded temperature for 2006 was 32°C and the mean relative humidity experienced was 75%^9^.

According to the manufacturer, Aspen Pharmaceuticals, stavudine should ideally be stored below 25°C in tightly closed containers^6^. Due to the stigma associated with the syndrome, patients in their attempts to hide the identity of the drug that they are taking from family, friends and even co-workers, often decant the drug into other more anonymous forms of packaging^10^.

Therefore, the aim of this study was to analyse the chemical stability of stavudine, in packaging other than the manufacturer's original packaging and under temperature and humidity conditions higher than those recommended by the manufacturer.

### The problem statement

According to the World Health Organisation^5^, stavudine must be stored in a well closed container protected from light. The manufacturer also recommends that stavudine be stored in tightly closed containers below 25°C^6^. However, literature has indicated that due to the stigma associated with the disease, stavudine may not always be stored by the patient under these conditions. Research has shown that stavudine can degrade to thymine under hydrolytic, oxidative and photolytic conditions. This identified degradation of stavudine may be especially problematic in sub-tropical and areas of high humidity, such as Durban, KwaZulu-Natal. Therefore, this study investigated the stability of stavudine in packaging other than that used by the manufacturer and under temperature and humidity conditions that are higher than those recommended by the manufacturer.

### Importance of this study

The stability of drug substance and product is essential for drug quality since it determines the efficacy of any drug or its dosage form^11^. Improving the quality of essential drugs, including ARV's, by strengthening quality control during storage, distribution and use is believed to ensure access to efficacious and safe drugs^12^. Thus the results obtained from this study, which will form the basis for recommendations for the storage of drugs during use, will promote the effective and safe ARV drug therapy.

### Pharmacology

Stavudine (d4T) (2’-3’-didehydro-2’-3’-dideoxythymidine), a thymidine nucleoside, is a reverse transcriptase inhibitor of the HIV. Upon administration, stavudine is phosphorylated by cellular kinases into its active triphosphate which inhibits HIV reverse transcriptase by competing with thymidine triphosphate (the natural substrate) for incorporation into the viral DNA and also causes chain termination. Thus any hindrance to the chemical stature and stability of the active drug will compromise the patient from achieving the desired therapeutic outcome i.e. reducing viral activity^13^.

## METHOD

Stavudine was received gratis from Aspen Pharmacare (South Africa) and thymine was purchased from Sigma Aldrich (Germany). All other chemicals were of analytical reagent grade. Since this study did not involve any interaction with humans or animals, ethical clearance was not required.

### HPLC analysis

All samples were analysed by HPLC. The analyses were carried out on a system consisting of a pump, a SPD-M20A prominence diode array detector, a SIL 20A prominence auto sampler and a degasser module (all equipment from Shimadzu, Japan). The data was acquired and processed by the use of LC Solution software version 12. Separations were achieved on an ACE C-18 column (4.6 X 150mm with particle size 5µ) using methanolwater in the ratio of 15:85. The injection volume was 10µl and the detection wavelength was 265nm.

### Identification of stavudine and thymine

**FIGURE 3:**
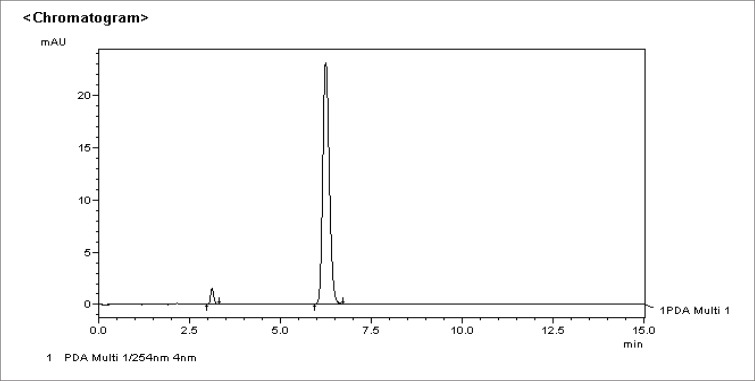
Chromatogram of stavudine standard 0.1mg/m [D4t was identified at an elution time of ± 6.25 minutes]

**FIGURE 4:**
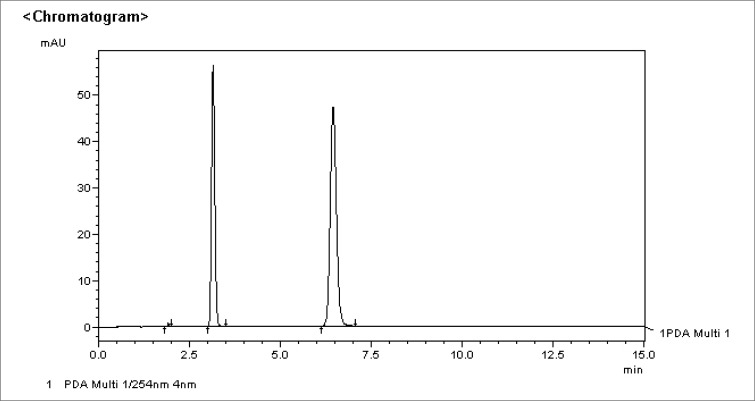
Chromatogram of thymine standard 0.5mg/ml [Thymine was identified at an elution time of ± 3.16 minutes.]

Using pure samples of stavudine and thymine, their respective elution times and chromatographic peaks were identified. These peaks are illustrated in Figure 3 and Figure 4 respectively.

### Calibration curve

A calibration curve for stavudine was obtained by plotting the mean chromatographic peak areas against their corresponding concentrations of stavudine.

### Degradation studies

All degradation studies were carried out at a concentration of 1mg/ml. A baseline reading giving the quantity of stavudine in the capsules was performed using stavudine capsules directly from the original manufacturer's container. The stavudine capsules were then placed in 2 different types of packaging, i.e. clear vials and plastic packets and then subjected to varying temperatures and humidity's, i.e. 30°C/75% RH (which represented the average humidity and temperature), and 50°C/ 95% RH (which represented the maximum temperature and relative humidity). At two week intervals capsules were withdrawn and subjected to an analysis via HPLC.

## RESULTS AND DISCUSSION

### Identification of stavudine and thymine

Using pure samples, the elution times for stavudine (d4T) and its degradation product, thymine, were obtained at +-6.25 minutes and +-3.16 minutes respectively.

### Correlation coefficient

Linearity in a concentration range from 0.03-0.1mg/ml was obtained, giving a correlation coefficient of 0.9639.

### Baseline results

**FIGURE 5:**
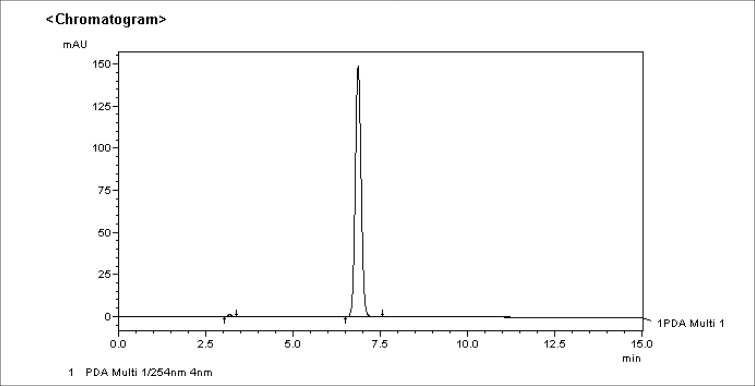
Chromatogram showing the stavudine peak on day zero

Figure 5 and Table 1 reported below shows the baseline results obtained for stavudine taken from the original package at day zero. The concentration of stavudine at day zero was found to be 99%.

**TABLE 1:** Stavudine concentration on day zero

	AREA	CONC	SD
**DAY 0**			
Sample (run 1)	2858320	0.989	0.001
Sample (run 2)	2865507	0.991	0.001
Average		0.990	
Appearance	NORMAL		

### Degradation behaviour

#### Storage conditions

**FIGURE 6:**
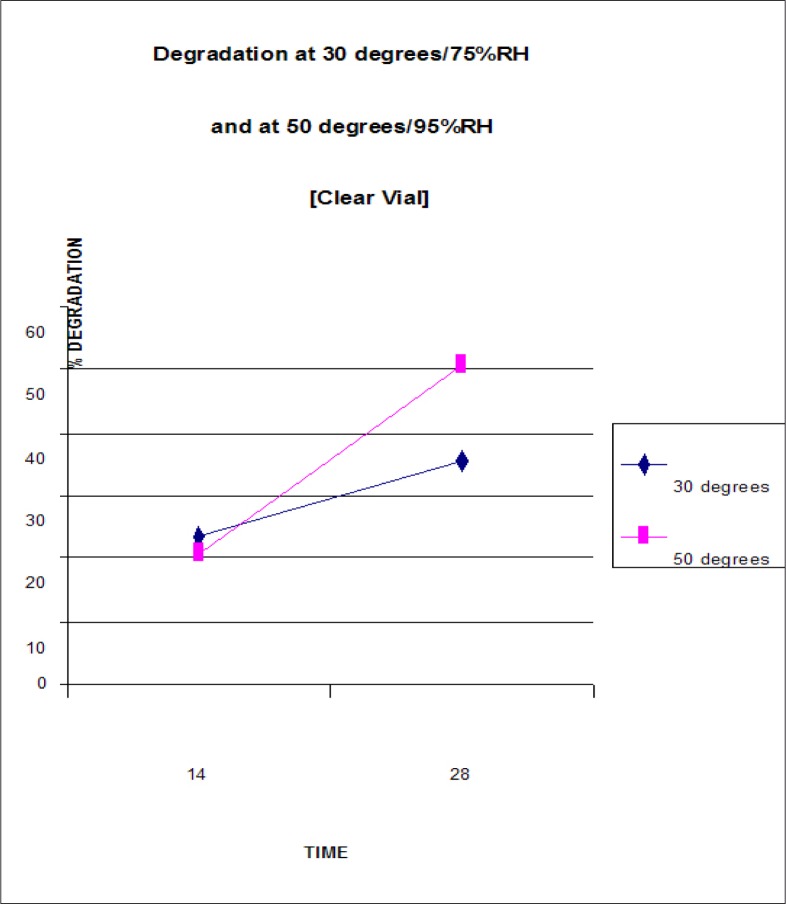
Stavudine degradation in capsules packaged in clear vials and stored at 300/75%RH and 500/95%RH

Table 2 and Figure 6 show the degradation results for the stavudine capsules packaged in clear vials and stored at 300/75%RH and 500/95%RH. An increase in the degradation of stavudine was observed under both storage conditions i.e. from 23.5% at day 14 to 35.7% at day 28 under the storage condition 300C/75%RH and from 20.6% at day 14 to 51% at day 28 under the storage condition 500C/95%RH.

**TABLE 2:** Stavudine degradation in capsules packaged in clear vials and stored at 300/75%RH and 500/95%RH

TIME			STORAGE CONDITIONS					
			
	30°C / 75% RH				50°C / 95%RH			
	
	AREA	CONC.	SD	% DEGRADATION	AREA	CONC.	SD	% DEGRADATION
**DAY 14**								
Sample(run1)	2213510	0.765	0		2295828*	0.794	0	
Sample(run2)	2208978	0.764	±0.001		2294538*	0.793	±0.001	
Average		0.765		23.5%		0.794		20.6%
Appearance	NORMAL				STICKY			
**DAY 28**								
Sample(run1)	1864951	0.644	±0.001		1420684*	0.490	0	
Sample(run2)	1857923	0.642	±0.001		1420292*	0.490	0	
Average		0.643		35.7%		0.490		51%
Appearance	STICKY				CHANGE IN COLOUR WITH CLUMPING			

**FIGURE 7:**
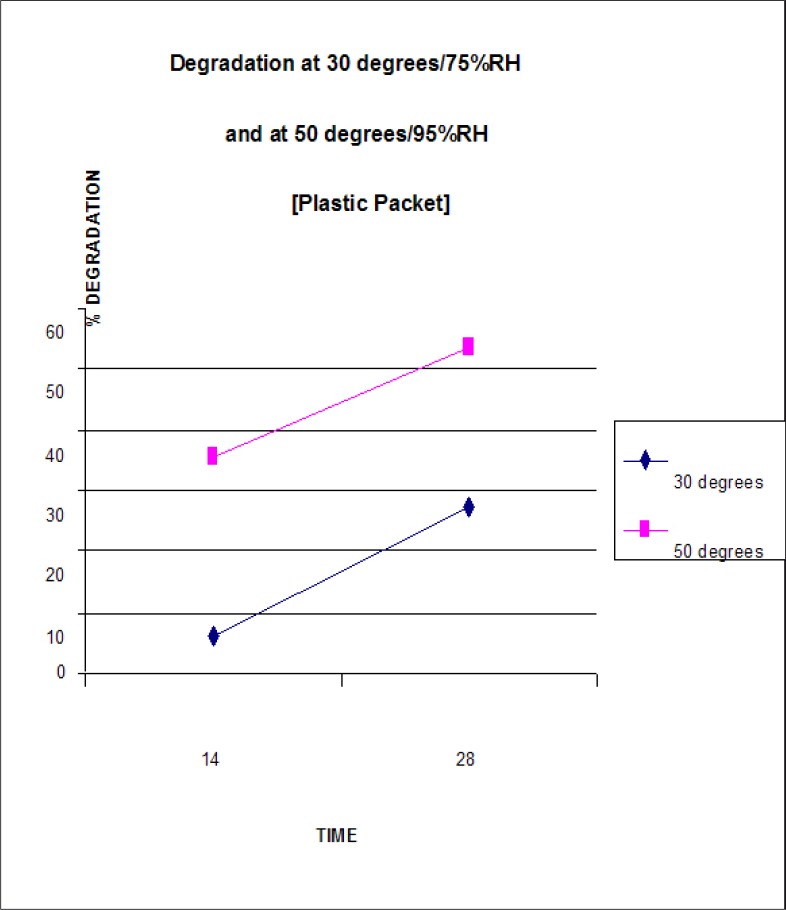
Stavudine degradation in capsules packaged in plastic packets and stored at 300/75%RH and 500/95%RH

Table 3 and Figure 7 show the degradation results for the stavudine capsules packaged in plastic packets and stored at 300/75%RH and 500/95%RH. An increase in the degradation of stavudine was observed under both storage conditions i.e. from 5.9% at day 14 to 27.4% at day 28 under the storage condition 300C/75%RH and from 35.7% at day 14 to 53.6% at day 28 under the storage condition 500C/95%RH.

**TABLE 3:** Stavudine degradation in capsules packaged in plastic packets and stored at 300/75%RH and 500/95RH

TIME			STORAGE CONDITIONS					
			
	30°C / 75% RH				50°C / 95%RH			
	
	AREA	CONC.	SD	% DEGRADATION	AREA	CONC.	SD	% DEGRADATION
**DAY 14**								
Sample(run1)	2718426	0.940	0		1862616*	0.644	±0.001	
Sample(run2)	2720633	0.941	±0.001		1858958*	0.642	±0.001	
Average		0.941		5.9%		0.643		35.7%
Appearance	NORMAL				STICKY			
**DAY28**								
Sample(run1)	2099744	0.726	0		1344286*	0.464	0	
Sample(run2)	2098285	0.725	±0.001		1342534*	0.463	±0.001	
Average		0.726		27.4%		0.464		53.6%
Appearance	STICKY				CHANGE IN COLOUR WITH CLUMPING			

#### Packaging

Tables 2 and 3 and Figures 6 and 7 show that greater degradation occurred in the stavudine packaged in the clear vial as opposed to the plastic packet on days 14 and 28 at 300C/75%RH. The explanation of this phenomenon may lie in the fact that the clear vial contained airspace which enabled increased moisture condensation leading to increased hydrolysis of the stavudine, while the plastic packet at the corresponding temperature and humidity contained little or no airspace leading to lower stavudine hydrolysis.

The opposite effect occurred at the storage condition 500C/95%RH, i.e. greater degradation occurred in the stavudine packaged in the plastic packet as opposed to the clear vial on days 14 and 28. The explanation for this phenomenon may lie in the fact that at the higher temperature of 500C, the plastic packet being less robust than the plastic vial afforded less environmental protection to the capsules. This coupled with the higher humidity of this test parameter, led to the increased degradation of stavudine.

### Conclusion

Previous research studies have shown that stavudine may degrade to thymine under hydrolytic, oxidative and photolytic conditions. This study has shown that stavudine, when packaged in clear vials and plastic packets and stored under conditions other than those recommended by the manufacturer, degrades significantly. The stigma associated with the disease condition HIV/AIDS, can cause patients to remove their medication from the original containers and place it in more anonymous packaging, exposing stavudine to less than optimal packaging and storage conditions. If the patient then ingests medication that contains less than the required dose of active ingredient, treatment failure can occur. This can contribute significantly to drug resistance and increased treatment costs. This study thus illustrates the importance of appropriate drug packaging and storage conditions.

Patients taking stavudine should be counselled about the proper storage of their medication and informed about the negative impact that improper storage can have on drug treatment. This study recommends that stavudine be dispensed in more anonymous, patient friendly original packs or alternately be packaged in blister packs.

## References

[1] SANAC HIV & AIDS and STI, National Strategic Plan 2007-2011 SANAC: Pretoria; 2007 p.7.

[2] South African Department of Health National Antiretroviral Treatment Guideline Pretoria: Government Printers; 2004.

[3] Gandhi RB, Bogardus JB, Bugay DE, et al. Pharmaceutical relationships of three solid state forms of stavudine. Int J Pharm. 2000;(2):201. 10.1016/s0378-5173(00)00419-1 10878328

[4] Ashenafi D, Chakraborti AK, Singh S. Mechanistic explanation to the variable degradation behaviour of stavudine and zidovudine under hydrolytic, oxidative and photolytic conditions. J Pharm Biomed Anal. 2004;(4):35. 15193743 10.1016/j.jpba.2004.03.007

[5] International Pharmacopoeia http://www.who.int/medicines/publications/pharmacopoeia/QAS_123rev2_Stavudine_mono_FINAL07.pdf.

[6] South African Electronic Package Inserts Aspen Stavudine [homepage on the Internet]. No date [cited 2008 February 25]. Available from: http://home.intekom.com/pharm/aspen-p/a-stavud.html.

[7] Ashenafi D, Sharda N, Singh B, Singh S. Establishment of inherent stability of stavudine and development of a validated stability-indicating HPLC assay method. J Pharm Biomed Anal. 2005;(5):37. 10.1016/j.jpba.2004.09.014 15862694

[8] Santoro MIRM, Taborianski AM, Singh AK, Kedor-Hackmann ERM. Stability-indicating methods for quantitative determination of zidovudine and stavudine in capsules. Química Nova. 2006;29.

[9] BBC BBC weather-Average conditions [homepage on the Internet]. No date [cited 2007 April 15]. Available from: http://www.bbc.co.uk/weather/world/city_guides/results.shtml?tt=TT000590

[10] Carol G, Isasi F, Bontempi JB, Eng E. Secret pills: HIV-Positive patients experiences taking antiretroviral therapy in North Carolina. J Gen Intern Med. 2002;14(4):318–329. 10.1521/aeap.14.5.318.23870 12212718

[11] Francis PA. Stability testing, A dire need. http://www.pharmbiz.com/article/detnews.asp?articleid=12382&sectionid=47.

[12] Binka J. National Drug Quality Control Consultancy Report. Ethiopia, USAID; 2004.

[13] Harvey RA, Cahmpe PC, Howland RD, Mycek MJ. Lippincott's Illustrated Reviews: Pharmacology 3rd ed New York: Lippincott and Wilkens; 2006.

